# Sex-biased differences in the effects of host individual, host population and environmental traits driving tick parasitism in red deer

**DOI:** 10.3389/fcimb.2013.00023

**Published:** 2013-06-27

**Authors:** Francisco Ruiz-Fons, Pelayo Acevedo, Raquel Sobrino, Joaquín Vicente, Yolanda Fierro, Isabel G. Fernández-de-Mera

**Affiliations:** ^1^Animal Health and Biotechnology Group (SaBio), Spanish National Wildlife Research Institute (IREC CSIC-UCLM-JCCM)Ciudad Real, Spain; ^2^Conservation Genetics and Wildlife Management Department, InBio Laboratório Associado, CIBIO, Centro de Investigação em Biodiversidade e Recursos Genéticos, Universidade do PortoVairão, Portugal; ^3^Yolfi PropertiesCiudad Real, Spain; ^4^Centro de Vigilancia Sanitaria Veterinaria (VISAVET), Departamento de Sanidad Animal, Universidad Complutense de MadridMadrid, Spain

**Keywords:** host-parasite, polygynous, cervidae, tick, sexual segregation

## Abstract

The interactions between host individual, host population, and environmental factors modulate parasite abundance in a given host population. Since adult exophilic ticks are highly aggregated in red deer (*Cervus elaphus*) and this ungulate exhibits significant sexual size dimorphism, life history traits and segregation, we hypothesized that tick parasitism on males and hinds would be differentially influenced by each of these factors. To test the hypothesis, ticks from 306 red deer—182 males and 124 females—were collected during 7 years in a red deer population in south-central Spain. By using generalized linear models, with a negative binomial error distribution and a logarithmic link function, we modeled tick abundance on deer with 20 potential predictors. Three models were developed: one for red deer males, another for hinds, and one combining data for males and females and including “sex” as factor. Our rationale was that if tick burdens on males and hinds relate to the explanatory factors in a differential way, it is not possible to precisely and accurately predict the tick burden on one sex using the model fitted on the other sex, or with the model that combines data from both sexes. Our results showed that deer males were the primary target for ticks, the weight of each factor differed between sexes, and each sex specific model was not able to accurately predict burdens on the animals of the other sex. That is, results support for sex-biased differences. The higher weight of host individual and population factors in the model for males show that intrinsic deer factors more strongly explain tick burden than environmental host-seeking tick abundance. In contrast, environmental variables predominated in the models explaining tick burdens in hinds.

## Introduction

Tick distribution in their hosts is frequently found to be highly aggregated in a few individuals within the host population, which determines that a few hosts are responsible for feeding large amounts of ticks (Shaw and Dobson, 1995; Shaw et al., 1998). This ecological feature of tick-host interactions greatly conditions the transmission of pathogens between ticks and their hosts (Perkins et al., 2003). The probability of tick-borne pathogen transmission at the tick-host interface largely depends on the burden of ticks feeding in a single infected individual, especially when co-feeding transmission is of great relevance for the epidemiology of the pathogen (Perkins et al., 2003). Thus, identifying factors driving tick-host relationships in each tick-host system is crucial to both prevent undesired effects on target and accidental hosts that may be highly susceptible to certain tick-borne pathogens and to reduce risks of transmission to humans of zoonotic pathogens.

Higher individual macroparasite burdens would be expected to be associated with lower immune capacity to fight against parasites (Vicente et al., 2007a), though recent studies link higher macroparasite burdens to host activity traits (Boyer et al., 2010), body mass (Kiffner et al., 2013) or to other effects linked to tick distribution in the environment (Calabrese et al., 2011). Since exophilic ticks are highly aggregated in the environment (Ruiz-Fons and Gilbert, 2010), the rate of host-tick effective contacts at a local spatial scale would consequently be expected to be higher for hosts displaying higher activity and higher body surface. However, many studies have dealt with the immunocompetence handicap hypothesis (Folstad and Karter, 1992) driving the burden of macroparasites in their hosts (Hughes and Randolph, 2001; Malo et al., 2009). The immunocompetence handicap hypothesis basically proposes that testosterone has a dual effect on males, enhancing expression of secondary sexual traits and depressing the immune system. Thus, better males could allow overexpression of sexual traits while overcoming negative effects related to immunocompetence reduction. Therefore, physical (morphology), ecological (behavior), and physiological (testosterone levels) factors have been considered as main drivers of parasitism in mammals (Moore and Wilson, 2002; Alzaga et al., 2009; Kiffner et al., 2013).

Sex-biased parasitism has been reported in many different host-parasite systems, often displaying a male-biased parasitism in highly dimorphic species (Moore and Wilson, 2002; Kiffner et al., 2013), especially those subjected to greater intraspecific competition for resources (Bacelar et al., 2011). Resource partitioning in self-maintenance, reproduction and defense against parasites is the result of a basic trade-off experienced by animals (Clutton-Brock et al., 1982). Mating system in polygynous mammals may carry over drastic resource allocation changes in individuals, especially in males, whose priorities in mating are more important than those related to immunocompetence (Rolff, 2002). Red deer (*Cervus elaphus*) males display a “live hard, die young” strategy (Carranza et al., 2004) in contrast to females that tend to allocate resources to self-maintenance, offspring rearing, and immunity, which has been deemed as one of the main sexual behavioral traits enhancing higher parasite loads in male red deer (Vicente et al., 2007a,b). Sex-related effects on tick burdens in mammals are controversial. Several authors described a clear sex-related effect on tick burden in white-tailed deer (*Odocoileus virginianus*; Schulze et al., 1984; Kitron et al., 1992; Schmidtmann et al., 1998), while recent studies in German roe deer (*Capreolus capreolus*) found no sex-related effect on tick burdens (Vor et al., 2010; Kiffner et al., 2011). The latter authors assume these host-species related differences in wild ruminants could be linked to sexual dimorphism, since dimorphism is higher in white-tailed than in roe deer, but it also could be linked to other factors related to sexual segregation.

Since adult exophilic ticks are highly aggregated in red deer and this wild ruminant exhibits a significant sexual size, resource allocation in immunity and behavioral dimorphism (e.g., Clutton-Brock et al., 1982), we hypothesized that parasitism by ticks—i.e., tick abundance on hosts—on each sex would be differentially influenced by host individual, host population and environmental factors. Predicting tick burdens in hinds with factors identified to drive tick burdens in males, and vice-versa, could be accurate only in the case of factors having the same weight on parasite burdens in both sexes. Otherwise, prediction of tick burdens, and hence identification of key hosts for tick-borne pathogens, would need to differentially consider host sex.

## Materials and methods

### Study area and host individual traits

The study area comprised a 900 ha hunting estate located in Ciudad Real province (south-central Spain: 38°55′N, 0°36′E; 600–850 m a. s. l.) of which 700 ha are dedicated to game rearing for hunting purposes, mostly Iberian red deer (*C. elaphus hispanicus*) and Eurasian wild boar (*Sus scrofa*) but also small numbers of mouflon (*Ovis aries musimon*) and aoudad (*Ammotragus lervia*). Orography in the estate is formed by hill chains (up to 100 m high) bordering three main valleys. Hills are covered by Mediterranean shrub ecosystem composed by scattered *Quercus ilex* trees and shrub dominated by *Cystus* spp. *Pistacia* spp., *Rosmarinus* spp., *Erica* spp., *Arbutus unedo*, and *Phyllirea* spp. Valleys are dedicated to grow seasonal cereal crops for game feeding. Climate is continental Mediterranean with cold winters and very hot and dry summers and rainfall—ranging 300–700 mm annually—is highly seasonal. Supplementary food (mixed cereal-leguminous pellets) is available *ad-libitum* along the year for deer on selective feeders located at the bottom of valleys (8 feeding points). Additionally, water supply is maintained all-over the year in eight water ponds distributed along riverbeds in valleys.

Over 7 years, from 2004 to 2010, hunter harvested red deer were surveyed for ticks immediately after being shot. The whole deer body was surveyed for ticks, which were counted and collected. Every tick from lowly parasitized animals (<30 ticks) was collected while a representative subsample of ticks were collected in highly parasitized individuals (>30 ticks). Every immature stage located was collected from deer carcasses. Collected ticks were identified to species level (Manilla, 1998; Estrada-Peña et al., 2004; Apanaskevich and Horak, 2008; Apanaskevich et al., 2008).

Every deer surveyed was subjected to a detailed necropsy to detect any lesion caused by macro or microparasites (e.g., tuberculosis-like lesions; see Vicente et al., 2006) in different organs, weighed, sexed, and biometrically characterized—total length, hind foot length, and thoracic perimeter (measured to the nearest 0.1 cm). During necropsies, spleen was weighed to the nearest 0.1 g and kidney fat index (KFI) was calculated as an estimation of body fat (Santos et al., 2013). Age was determined for young individuals (<2 years old) based on tooth eruption patterns (Sáenz de Buruaga et al., 1991) and by incisor 1 sectioning in >2 year old animals (Klevezal and Kleinenberg, 1967). Deer age was categorized into 5 classes: (1) fawns (0–1 year old); (2) yearlings (1–2 years old); (3) subadults (2–3 years old); (4) adults (4–10 years old); and (5) old (>10 years old). Maximum recorded age was 23 years. Individual host data throughout sex and age class is shown in Table 1.

**Table 1:** **Average values, associated standard error and range (within brackets) of host individual variables [total length (TL; cms), thoracic perimeter (TP; cms), hind foot length (HF; cms), and kidney fat index (KFI; %)] throughout sex and age class of studied deer**.

**Sex**	**Age class**	**TL**	**TP**	**HF**	**KFI**
Male	Fawn	133.8 ± 2.7 (104–149)	93.4 ± 2.6 (68–115)	45.3 ± 0.9 (34–51)	88.0 ± 21.3 (9.5–385.5)
	Yearling	165.8 ± 1.3 (149–178)	115.0 ± 1.9 (101–132)	51.8 ± 0.4 (48–56)	4.3 ± 6.4 (4.6–107.7)
	Subadult	174.9 ± 2.4 (161–187)	115.9 ± 1.7 (108–127)	53.1 ± 1.7 (49–62)	41.6 ± 7.8 (15.2–85.6)
	Adult	187.7 ± 1.1 (158–213)	125.8 ± 0.7 (111–155)	53.3 ± 0.2 (48–61)	65.7 ± 8.5 (4.9–455.3)
	Old	188.8 ± 2.6 (174–203)	125.0 ± 1.9 (116–134)	52.8 ± 0.7 (49–56)	48.9 ± 14.0 (7.5–106.4)
	Subtotal male	178.1 ± 1.5 (104–213)	120.2 ± 1.0 (68–155)	52.2 ± 0.3 (34–62)	63.0 ± 6.5 (4.6–455.3)
Female	Fawn	128.5 ± 3.5 (95–152)	86.1 ± 2.6 (64–106)	44.3 ± 0.8 (35–49)	78.0 ± 19.3 (7.4–247.0)
	Yearling	148.4 ± 2.9 (130–161)	108.0 ± 6.5 (88–162)	49.1 ± 0.7 (45–53)	122.2 ± 23.2 (61.0–241.0)
	Subadult	160.6 ± 2.3 (147–174)	105.2 ± 2.0 (91–114)	49.3 ± 0.3 (47–51)	74.7 ± 17.6 (20.0–232.0)
	Adult	162.4 ± 1.1 (133–184)	110.3 ± 0.9 (89–130)	48.7 ± 0.2 (43–53)	93.8 ± 8.4 (2.4–263.5)
	Old	166.6 ± 1.7 (160–178)	110.2 ± 1.7 (104–120)	48.7 ± 0.4 (47–51)	119.7 ± 25.0 (30.9–283.9)
	Subtotal female	157.2 ± 1.3 (95–184)	106.0 ± 1.0 (64–130)	48.2 ± 0.2 (35–53)	94.4 ± 6.6 (2.4–283.9)

### Host population traits

Host abundance is a key factor influencing host-seeking tick burdens in the environment at local geographic scales that could greatly condition tick burden in individual hosts (Ruiz-Fons et al., 2012). At the short time-scale (i.e., a year) the influence of host abundance on tick environmental abundance is difficult to measure since individual ticks may take up to several years to complete their life cycle. However, at the long-time scale changes in key host availability between years may be reflected in changes in host-seeking tick abundance. Wild ungulates are key hosts for adults of the predominant tick species in the estate (*Hyalomma lusitanicum* and *Rhipicephalus bursa*; see Ruiz-Fons et al., 2006) so annual censuses for the most abundant ungulate species in the estate, that is, red deer and wild boar, were used as predictors for tick burden models. The effect of host abundance in previous years on current tick burdens was tested by considering deer and wild boar abundance in years *t*-1 and *t*-2 (see Ostfeld et al., 2006). Censuses were performed by experienced observers (gamekeepers) who counted individuals approaching feeders at the bottom of the valleys (total counts) during the red deer rut season. For further details on the census procedure see Rodríguez-Hidalgo et al. (2010).

### Environmental variables

Climatic conditions (e.g., temperature and hydric stress) greatly condition tick phenology, activity, and survival (Estrada-Peña et al., 2011). Adult tick burden in an individual host at a given time is a function of the ticks encountered by the individual within a two week period since this is the average time adult *Hyalomma* ticks remain feeding in their host (Estrada-Peña et al., 2011). Thus, meteorological data at the short time scale, i.e., in 30 days before each animal was surveyed, were considered as a proxy of climatic constraints of tick activity. Considering a 30 days period aimed to buffer the occurrence of any stochastic meteorological event that could have momentarily affected tick questing behavior and consequently tick burdens on hosts. Meteorological data—temperature and precipitation—on a daily basis were obtained from a meteorological station (Spanish Meteorological Agency reference station 4210E; http://www.aemet.es) located in the study hunting estate (Table 2). The actual evapotranspiration (AET)—a measure of hydric stress experienced by ticks in its off-host period—was calculated on the basis of temperature and precipitation data using the formula proposed by Turc (1954), as follows:

$$\text{AET}=\frac{P}{\sqrt{0.9}+\left(\frac{P^{2}}{L^{2}}\right)}$$

where “*P*” is accumulated precipitation in mm and “*L*” is defined by:

$$L=300\times 25t+0.05t^{3}$$

being “*t*” the mean temperature in °C.

**Table 2:** **Deer, wild boar, total ungulate (deer + boar + mouflon + aoudad) counts, and average values of climatic variables (and associated standard error within brackets) associated to deer sampling date in the hunting estate throughout year**.

**Year**	**Deer_C**	**Wild boar_C**	**Tot_Ung_C**	**AvT_M^a^**	**AP_M^b^**	**AET_M^c^**
2002	363	160	600	NA	NA	NA
2003	365	60	504	NA	NA	NA
2004	286	40	395	20.1 (1.2)	8.0 (2.1)	0.97 (6.4 × 10^−3^)
2005	400	140	626	22.0 (1.2)	8.3 (3.2)	0.25 (7.3 × 10^−2^)
2006	392	100	559	15.3 (1.2)	47.8 (5.2)	0.99 (4.8 × 10^−4^)
2007	425	200	693	16.5 (1.0)	47.3 (3.5)	0.99 (1.0 × 10^−4^)
2008	418	150	636	13.0 (0.8)	68.2 (4.7)	0.93 (4.2 × 10^−2^)
2009	434	16	514	20.4 (0.5)	31.7 (1.8)	0.89 (3.2 × 10^−2^)
2010	332	48	458	8.9 (1.1)	151.6 (9.4)	0.99 (8.2 × 10^−6^)
Average	379.4	101.5	553.8	—	—	—

a AvT_M, average mean daily temperature (°C) values of 30 days before sampling (bs);

b AP_M, accumulated precipitation (mm) of 30 days bs;

c AET_M, actual evapotranspiration (mm) of 30 days bs. NA, Not applicable.

### Statistical modelling and analytical design

For descriptive analyses of parasitization rates the statistical uncertainty was assessed by calculating the 95% confidence interval for each of the proportions according to the expression 95%C.I. = 1.96[*p*(1−p)/*n*]^1/2^ (where “*p*” is the proportion in its unitary value and “*n*” is the sample size) and expressed in percentage.

Using an inductive approach we quantified the effect of the main factors able to explain tick burdens on red deer, at individual level. Predictors were considered in generalized linear models with a negative binomial distribution and a logarithmic link function (Cameron and Trivedi, 1998), and the final models (three, see below) were obtained using a forwards-backwards stepwise procedure based on Akaike Information Criteria (AIC; Akaike, 1974). We opted for the negative binomial distribution due to high levels of overdispersion in the data when models were fitted with Poisson distributions. The multicolineality among predictors included in the final models was assessed using predictor's variance inflation factor (VIF). VIFs were calculated—for each predictor and model—as the inverse of the coefficient of non-determination for a regression of a given predictor on all others (see Zuur et al., 2010).

Because we were interested if tick-burdens were affected differentially in male and female deer, we developed three models: a model for red deer males, a model for hinds, and, finally, a model combining data for males and hinds and including “sex” as factor. If parasitization by ticks on red deer males and hinds responded to the explanatory factors in a differential way, it would be not possible to precisely and accurately predict the tick burden on hinds using the model fitted on males (and/or vice-versa), or with the model that combines data for males and hinds. However, if parasite loads on males and hinds similarly responded to the explanatory factors, then any of the independent models could precisely determine the rates of either sex, and in this occasion better adjust terms and more accurate predictions could be attained with the model carried out by combining data from males and hinds than with the independent model for each sex. Two analytical procedures were used in order to compare the model in the previous terms: variation partitioning and cross-validation.

**Figure A1:**
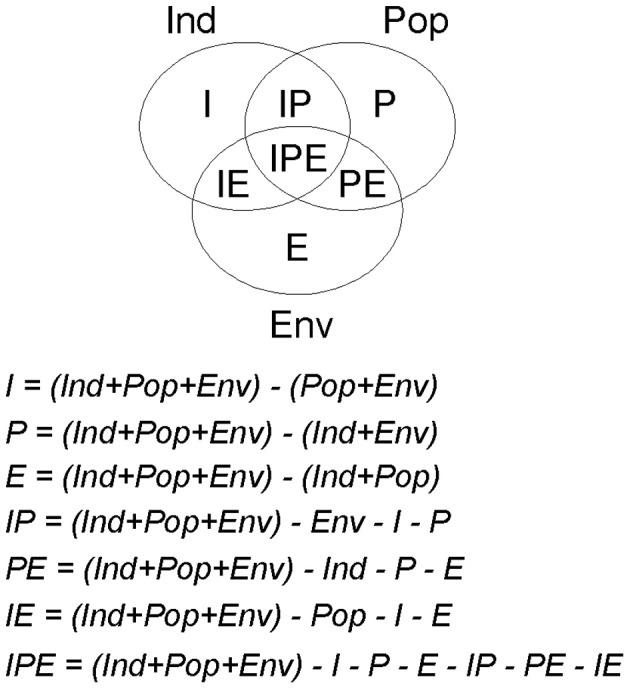
**Scheme of the parts in which the deviance explained by a final model can be split by variation partitioning procedures, and the subtraction rules used for this purpose**. For the variation partitioning we first determined the total amount of deviance explained by the final model, and secondly we developed partial models, i.e., the models adjusted independently with the predictors related to each factor (individual host: Ind; host population: Pop; and environment: Env), as well as with those of each pair of factors (Ind + Pop, Ind + Env, and Pop + Env), and estimated the amounts of deviance explained by each of these six partial models. The values of the deviance explained for the final model (Ind + Pop + Env) and for the partial models were used in the following subtraction rules.

Variation partitioning procedures (see Borcard et al., 1992) were used to estimate the variation of the final models explained independently by each factor (pure effects) and the variation explained simultaneously by two or more factors (overlaid effects; see Figure A1). Note that a factor is a group of related-predictors; in this study three factors: individual host, host population and environment. For this purpose, we determined the total amount of deviance explained by the final model. Subsequently, we developed the partial models, i.e., models adjusted independently with the predictors related to each factor (individual host: Ind, host population: Pop, and environment: Env), as well as with those of each pair of factors (Ind + Pop, Ind + Env, and Pop + Env), and estimated the amounts of deviance explained by each of these six partial models. Values of the deviance explained by the final model (Ind + Pop + Env) and those explained by the partial models were subjected to subtraction rules in order to split up the different sections of the explained variation (see Alzaga et al., 2009). A complete scheme of each part of deviance and the subtraction rules used for their determination, is reported in Appendix. Briefly, the proportion of variation explained exclusively—independently of the other factors—by the individual host, for instance, was obtained with the following subtraction rule: I = (Ind + Pop + Env) − (Pop + Env); the proportions explained exclusively by the other factors were obtained in a similar way. The amount of variation attributable to the intersection of two factors (e.g., individual host and host population) was obtained with the subtraction rule: IP = (Ind + Pop + Env) − Ind − P − E; where P is the explained variation by the pure effect of host population and E is the pure effect of environment. The amount of variation attributable to the intersections between individual and environmental factors (IE) and between population and environmental factors (PE) were calculated in a similar way, and the amount attributable to the intersections between the three factors together (IPE) was obtained with the subtraction rule: IPE = (Ind + Pop + Env) − E − P − I − EP − IP − EI. Therefore, we determined a value for each part of deviance explained and knew how much corresponded to its pure effect and how much to intersections between two or three factors. This procedure was carried out on each of the three final models. The proportions of explained deviance for each factor were standardized to make them comparable among models; for this purpose they were expressed in relation to the proportion of deviance explained for the final model (e.g., Alzaga et al., 2009; Pérez-Ramírez et al., 2012).

Cross-validation is a procedure for assessing how the results of a statistical model can be generalized to an independent data set (Picard and Cook, 1984). Under our analytical design, we are interested in how the results of the model developed on the dataset for a given sex can be used to explain variation in the response variable on the dataset for another sex (validation dataset). Similarly, we assessed the performance of the model developed by combining data for males and hinds, which was calibrated using an 70% random sample (training dataset) and was validated against the remaining 30% of the data (validation dataset). On each dataset and under this crossed framework, we binned predictions from the model into 10 evenly sized intervals of increasing predicted burdens. Assessment was carried out by plotting the mean observed against predicted abundance, in each interval on the validation datasets (see Pearce and Ferrier, 2000). The basic premise is that as the burdens predicted by the model increase (e.g., model for males), there should be a similar increase in the observed burdens in the validation dataset (in this case, on hinds dataset).

Statistical analyses were carried out in R 2.15.2 (R Core Team, 2012). The “MASS” library was used for model development (Venables and Ripley, 2002), the “HH” package for the VIF analyses (Heiberger, 2012), and the “ggplot2” package for the calibration plots (Wickham, 2009).

## Results

Tick data from 306 red deer—182 males and 124 females—were gathered for the 7 years of study (average deer no./year: 25.5; range: 12–64; Table 3). The 59.5% (95%CI: 54.0–65.0) of deer were parasitized by ticks, the major part only by adults (59.2%; 95%CI: 53.7–64.7). Out of the 4009 ticks counted on deer, 1772 were collected (1761 adults and 11 nymphs). Adults belonged mainly to *Hy. lusitanicum* (*n* = 1750; 98.8%), *Rh. bursa* (*n* = 9; 0.5%), *Rh. sanguineus* (*n* = 1; 0.05%), and *Dermacentor marginatus* (*n* = 1; 0.05%) and nymphs belonged to *Hy. lusitanicum* (*n* = 9; 0.5%) and *Rh. bursa* (*n* = 2; 0.1%). Annual average adult tick abundance per deer experienced a decrease along study years (Table 4).

**Table 3:** **Data on the number of tick parasitized deer (PosT) with respect the total number (N) of analyzed deer throughout sex and age class**.

**Sex**	**Age class**	**PosT/N**	**PrevT**	**Col-AvT**	**Cou-AvT**	**Col-AvA**	**Cou-AvA**
Male	Fawn	2/20	10.0	0.2 (1–2)	0.2 (0–2)	0.1 (0–2)	0.1 (0–2)
	Yearling	21/24	87.5	7.7 (0–49)	14.5 (0–50)	7.5 (0–49)	14.4 (0–50)
	Subadult	10/11	90.9	10.7 (0–36)	15.6 (0–60)	10.1 (0–36)	14.8 (0–60)
	Adult	104/118	88.1	9.5 (0–67)	24.0 (0–125)	9.2 (0–67)	23.6 (0–125)
	Old	9/9	100.0	17 (5–47)	39.3 (0–140)	16.9 (5–47)	39.0 (0–140)
	Subtotal male	146/182	80.2	8.6 (0–67)	20.4 (0–140)	8.4 (0–67)	20.0 (0–140)
Female	Fawn	2/16	12.5	0.3 (0–2)	0.3 (0–2)	0.2 (0–2)	0.2 (0–2)
	Yearling	2/10	20.0	1.2 (0–11)	1.2 (0–11)	1.2 (0–11)	1.2 (0–11)
	Subadult	4/13	30.8	1.1 (0–6)	1.7 (0–12)	1.1 (0–6)	1.7 (0–12)
	Adult	22/72	30.6	1.4 (0–21)	2.2 (0–36)	1.4 (0–21)	2.2 (0–36)
	Old	6/12	50.0	5.5 (0–25)	8.5 (0–49)	5.5 (0–25)	8.5 (0–49)
	Unknown	0/1	0.0	0.0 (0–0)	0.0 (0–0)	0.0 (0–0)	0.0 (0–0)
	Subtotal female	36/124	29.0	1.6 (0–25)	2.4 (0–49)	1.6 (0–25)	2.4 (0–49)
TOTAL		182/306	59.5	5.8 (0–67)	13.1 (0–140)	5.6 (0–67)	12.9 (0–140)

Average number of ticks/deer collected (Col_AvT) and counted (Cou_AvT) as well as collected (Col_AvA) and counted (Cou_AvA) adult ticks are displayed.

Values within brackets represent minimum and maximum collected and counted ticks and adult ticks per deer. The female with unknown age was not considered for modeling purposes.

**Table 4:** **Average number of ticks/deer collected (Col_AvT) and counted (Cou_AvT) and average number of adult ticks/deer collected (Col_AvA) and counted (Cou_AvA) throughout year and season**.

**Year**	**Season**	**N**	**Col_AvT**	**Cou_AvT**	**Col_AvA**	**Cou_AvA**
2004	Winter	0	NS^a^	NS	NS	NS
	Spring	0	NS	NS	NS	NS
	Summer	3	14.3 (6–19)	29.3 (9–60)	14.0 (6–19)	28.2 (9–57)
	Autumn	9	17.2 (4–47)	39.4 (4–140)	17.0 (2–47)	39.2 (2–140)
Subtotal 2004		12	16.5 (4–47)	36.9 (4–140)	16.3 (2–47)	36.5 (2–140)
2005	Winter	2	0.0 (0–0)	0.0 (0–0)	0.0 (0–0)	0.0 (0–0)
	Spring	1	25.0 (25–25)	49.0 (49–49)	25.0 (25–25)	49.0 (49–49)
	Summer	24	6.5 (0–49)	14.0 (0–50)	6.5 (0–49)	13.9 (0–50)
	Autumn	9	8.6 (0–31)	18.2 (0–48)	8.6 (0–31)	18.2 (0–48)
Subtotal 2005		36	7.2 (0–49)	15.2 (0–50)	7.1 (0–49)	15.2 (0–50)
2006	Winter	20	4.7 (0–22)	5.7 (0–28)	4.4 (0–22)	5.3 (0–28)
	Spring	0	NS	NS	NS	NS
	Summer	17	12.9 (0–67)	23.4 (0–125)	11.1 (0–67)	20.7 (0–125)
	Autumn	19	8.2 (0–27)	28.5 (0–120)	8.2 (0–27)	28.5 (0–120)
Subtotal 2006		56	8.4 (0–67)	18.8 (0–125)	7.7 (0–67)	17.8 (0–125)
2007	Winter	25	4.6 (0–14)	18.3 (0–80)	4.6 (0–14)	18.2 (0–80)
	Spring	1	0.0 (0–0)	0.0 (0–0)	0.0 (0–0)	0.0 (0–0)
	Summer	10	11.4 (0–24)	21.7 (0–60)	10.9 (0–21)	21.1 (0–60)
	Autumn	28	4.7 (0–28)	13.4 (0–80)	4.7 (0–28)	13.4 (0–80)
Subtotal 2007		64	5.7 (0–28)	16.4 (0–80)	5.6 (0–28)	16.3 (0–80)
2008	Winter	9	2.6 (0–15)	2.8 (0–16)	2.6 (0–15)	2.8 (0–16)
	Spring	0	NS	NS	NS	NS
	Summer	1	5.0 (5–5)	12.0 (12–12)	5.0 (5–5)	12.0 (12–12)
	Autumn	30	2.5 (0–25)	3.7 (0–28)	2.5 (0–25)	3.7 (0–28)
Subtotal 2008		40	2.6 (0–25)	3.7 (0–28)	2.6 (0–25)	3.7 (0–28)
2009	Winter	1	0.0 (0–0)	0.0 (0–0)	0.0 (0–0)	0.0 (0–0)
	Spring	0	NS	NS	NS	NS
	Summer	6	9.7 (1–23)	13.2 (1–40)	9.5 (1–23)	13.0 (1–40)
	Autumn	53	4.7 (0–19)	11.3 (0–80)	4.7 (0–19)	11.4 (0–80)
Subtotal 2009		60	5.1 (0–23)	11.3 (0–80)	5.1 (0–23)	11.4 (0–80)
2010	Winter	30	0.0 (0–0)	0.0 (0–0)	0.0 (0–0)	0.0 (0–0)
	Spring	0	NS	NS	NS	NS
	Summer	2	12.0 (11–13)	12.0 (11–13)	12.0 (11–13)	12.0 (11–13)
	Autumn	6	8.5 (3–21)	11.2 (4–30)	8.5 (3–21)	11.2 (4–30)
Subtotal 2010		38	2.0 (0–21)	2.4 (0–30)	2.0 (0–21)	2.4 (0–30)
TOTAL	Winter	87	2.7 (0–22)	6.9 (0–80)	2.3 (0–22)	6.7 (0–80)
	Spring	2	12.5 (0–25)	24.5 (0–49)	12.5 (0–25)	24.5 (0–49)
	Summer	63	9.8 (0–67)	18.3 (0–125)	9.2 (0–67)	17.4 (0–125)
	Autumn	154	5.8 (0–47)	14.4 (0–140)	5.8 (0–47)	14.4 (0–140)

a NS, No samples.

Predictors included in the three final models are summarized in Table 5. VIFs obtained for the predictors included in final models showed that no biased predictions are expected due to collinearity-derived problems (VIFs < 2.21, <2.32, and <1.99, for the model for males, for hinds, and for males and hinds, respectively). A higher amount of deviance was explained for the model of males (53.97%) than for the other models (46.26% and 50.76%, for the model of hinds and the model of males and hinds, respectively). When data for males and females were considered in a model, “sex” was a relevant predictor and a significantly higher number of ticks was detected on males than on hinds (see also Table 3). The observed increasing tick burden with deer age was evidenced in both males and females (Table 5). Predictors related to the three considered factors (i.e., individual host, host population and environment) were selected for the three final models; but according to test-values, the relevance of the predictors varied among the models (Table 5).

**Table 5:** Statistical parameters (coefficient/test-value and significance: ns: non-significant, #0.1, ^*^0.05, ^**^0.01, and ^***^0.001) of the generalized lineal models (negative binomial error distribution and logarithmic link function) carried out to predict tick burden on red deer.

**Predictor (factor)**	**Model for males**	**Model for hinds**	**Model for males and hinds**
TL (Ind)	0.0213/3.58^***^	0.0316/1.20 ns	0.0400/4.45^**^
AvT_M (Env)	0.0873/6.74^***^	0.1376/3.63^***^	0.0962/6.54^***^
Age class (Ind)	0.6245/5.18^***^	0.5950/1.70#	0.5337/3.54^***^
Year (Env)	−0.4869/−7.16^***^		
Deer_C (Pop)	0.0158/5.94^***^		0.0083/3.22^**^
Deer_C t-2 (Pop)		0.0097/1.84#	
ETA_M (Env)	1.1880/4.40^***^		1.7497/4.63^***^
KFI (Ind)	−0.0033/−3.19^**^		−0.0032/−2.50^*^
AP_M (Env)	−0.0067/−2.49^*^	−0.0313/−3.64^***^	−0.0189/−6.47^***^
Wild boar_C t-2 (Pop)			−0.0101/5.42^***^
Sex(females) (Ind)^a^			−1.5922^a^/−5.19^***^
Intercept	965.5163/7.11^***^	−11.5223/−2.77^**^	−10.6220/−6.35^***^

Individual host (Ind), host population (Pop), and environmental (Env) factors; predictors coded as in Tables 1–2.

a Coefficient for females in relation to males.

**Figure 1:**
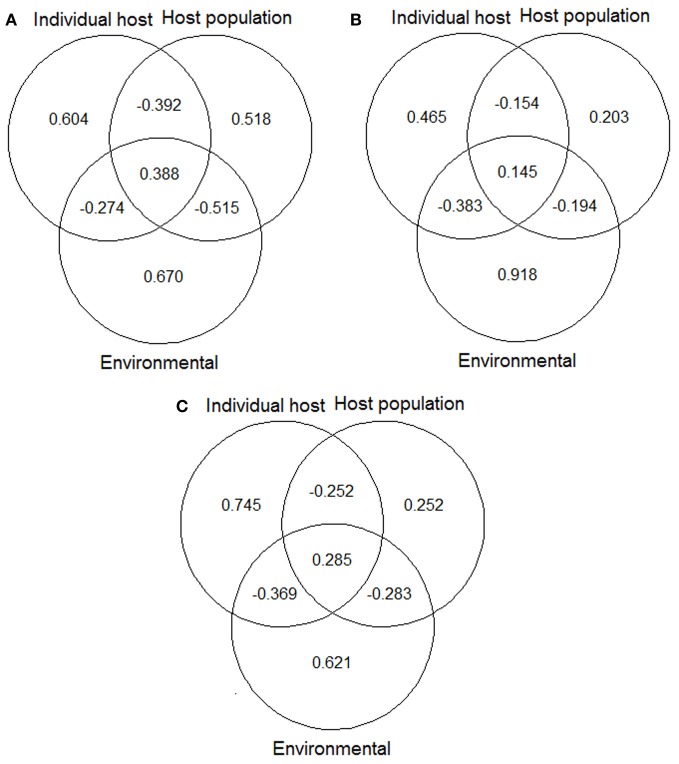
**Variation partitioning of the deviance explained by final models: (A) model for red deer males; (B) model for hinds; and (C) model for males and hinds**. Values shown in the diagrams are the proportions of variation of each final model that can be explained exclusively by individual host, host population and environmental factors, and by the combined effect of these factors. See Table 5 for details about predictors included in each of the abovementioned models/factors. The “VarPart” function was used for producing the plots (Barbosa et al., 2013).

Variation partitioning procedures showed that the amount of variation explained by the pure factors and the overlaid effects were quite different among the three models, mainly between the model for males and that for hinds (Figure 1). In the model for males, individual host and host population factors explained a higher amount of variation than in the model for hinds. For the hind model most of variation could be explained by the environmental factor. Finally, the model combining data from males and hinds showed an intermediate situation between the independent models for each sex, with a similar amount of variation explained by the host population factor than in the model of hinds, and a similar amount of variation explained by the environmental factor than in the model of males.

**Figure 2:**
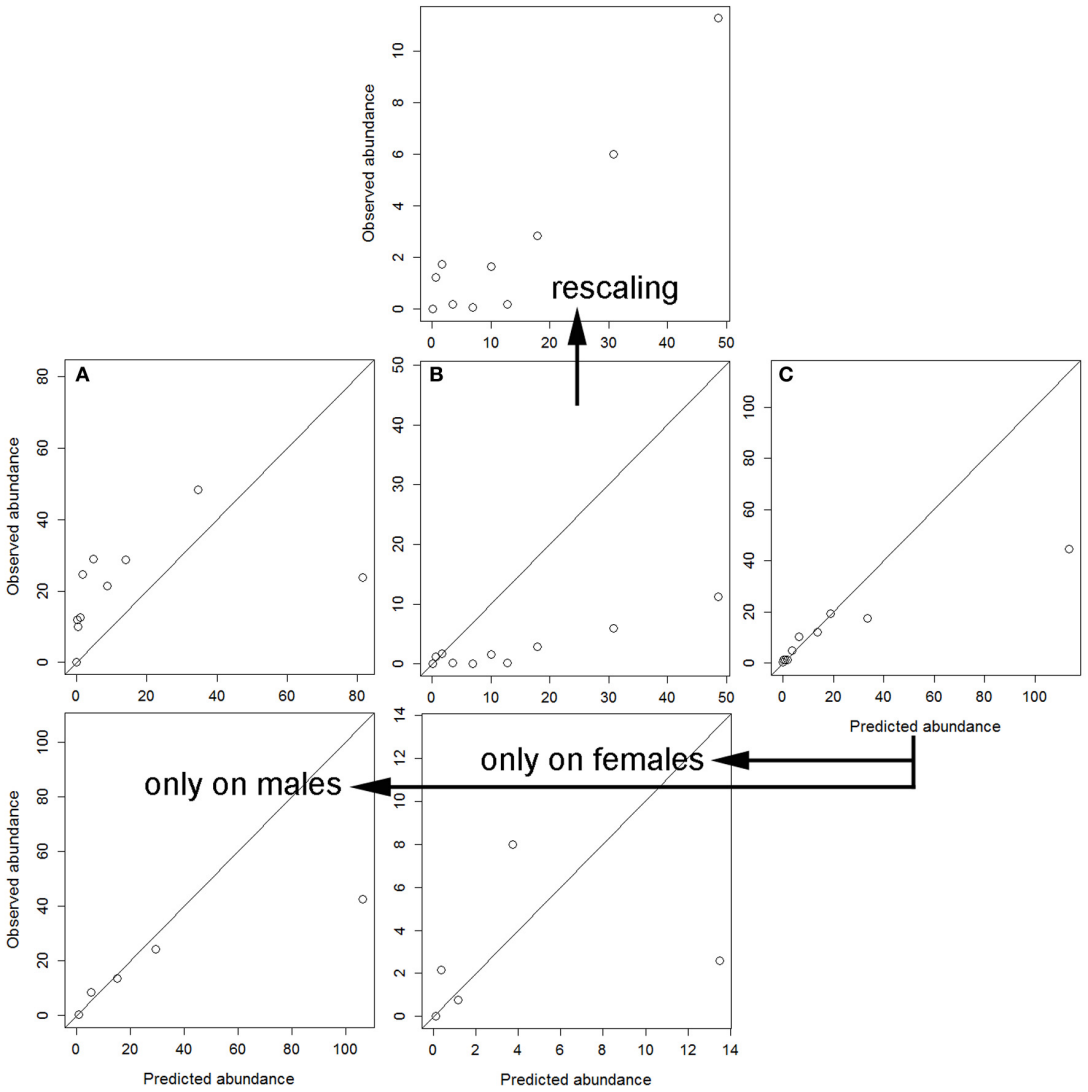
**Calibration's assessment of the three models (see Table 5) under a cross-validation procedure: (A) predictions from the model for hinds on the dataset for males; (B) predictions from the model for males on the dataset for hinds (also rescaling the observed abundance axis); and (C) predictions from the model for males and hinds on the validation dataset, also independently for males and females (only five intervals were used in these last cases due to sample size)**.

Finally, cross-validation showed that the independent models for each sex were not able to accurately explain the parasitization rate on the other sex (Figure 2). To this respect, the worst performance was obtained when the model for hinds was applied to the dataset of males (Figure 2A). The model for males precisely, but not accurately, explained tick parasitization on hinds, mainly for individuals with higher parasitization rates (Figure 2B); the model was precise because the observed abundance monotonically increased with predicted abundance (mainly for the higher intervals of predicted abundance), and it was not accurate because predictions overestimated the observed abundances. Finally, a model was adjusted by pooling data for males and females, and this model again overestimated the higher intervals of predicted abundance (Figure 2C). This combined model was closer to the response of males than to that of hinds.

## Discussion

Identification of factors driving tick parasitism on hosts has been a relevant issue in ecology (Moore and Wilson, 2002; Boyer et al., 2010; Calabrese et al., 2011), and is currently a relevant topic in tick-borne disease epidemiology (Perkins et al., 2003; Ruiz-Fons et al., 2012). Most studies have centered attention to small mammals (Brunner and Ostfeld, 2008; Alzaga et al., 2009; Boyer et al., 2010) and few attention has been paid to large mammals. Large mammals—such as red deer—are key hosts for many epidemiologically relevant tick species (Ruiz-Fons and Gilbert, 2010; Ruiz-Fons et al., 2012), especially in Mediterranean environments (Ruiz-Fons et al., 2006). In south-central Spain, red deer are abundant (Acevedo et al., 2008) and have experienced a notable increase in the last three decades (Apollonio et al., 2010), which would have consequences for tick ecology and for zoonotic tick-borne pathogen epidemiology (e.g., *Anaplasma phagocytophilum*—(de la Fuente et al., 2005)—or Crimean-Congo haemorrhagic fever virus—Estrada-Peña et al., 2013). Mating system (Miller et al., 2007), sexual dimorphism (Moore and Wilson, 2002), intraspecific competition (Bacelar et al., 2011), space use (Boyer et al., 2010), testosterone levels (Hughes and Randolph, 2001), and even effects of environmental host-tick interactions (Calabrese et al., 2011), have been proposed as relevant factors driving sex-biased parasitism in mammals. Identifying factors driving tick-deer interactions at the individual level is thus a crucial issue for efficiently preventing and controlling tick-borne disease risks. In this study, deer males were evidenced as primary targets of exophilic ticks, mainly *Hyalomma* spp., and we showed that parasitism on each sex was differentially determined by host individual, host population and environmental factors.

### Differential drivers of tick parasitism in males and hinds

Even when tick burdens on males and hinds were modeled with the same set of predictors, each sex specific model was not able to accurately predict tick burdens on animals of the other sex (Figure 2). This cross-validation procedure allows us to suggest that tick burden in red deer are driven by different traits on males and hinds (see also Vicente et al., 2007a). In addition, the model for males was able to predict—with moderate precision, better for higher tick abundance—burdens on hinds. This was likely due to the over-dominance of the environmental factor in the model for hinds. Parasites benefit from situations in which hosts are not in good conditions (Murray et al., 1998). Variations in condition along the year in food supplemented populations are lower in hinds than in males (Santos et al., 2013; see also Rodríguez-Hidalgo et al., 2010), since the latter are strongly affected by the rut period. Likely, this can be one of the reasons by which tick parasitism on males is more dependent on intrinsic factors that parasitism on hinds. Finally, the model combining data from males and females was better adjusted to variation in males than in hinds, evidencing again that parasitism in hinds is likely a simplification of the process in males.

Differential effects of host individual, host population and environmental factors in relation to the life cycle of parasites were evidenced in other mammal species (Alzaga et al., 2009). In European hare (*Lepus europaeus*), Alzaga et al. (2009) showed that the individual factor was the more explicative factor of tick burdens, followed by environmental and host population factors. In our study, we found relevant differences in the effects of each factor in relation to host sex, but in male and global models the effect of the individual factor was not clearly inferior to the others. Differences between the study on hares and the present study are likely related to the ecology of the host-tick system, i.e., ecological traits of host species but also of ticks, since different tick species were found parasitizing European hare and deer. In our study model, individual host and host population factors were more relevant for males than for hinds. In contrast, the environmental factor was more important explaining burdens on hinds. Potential reasons mediating these sex-biased differences are discussed below.

### Host individual factor driving tick parasitism in red deer

Individual predictors such as size and age were positively related to tick abundance in red deer, while KFI was negatively related in the model for males. Size—measured by total length—and body mass were highly correlated in our data set (Spearman's rho = 0.904, *p* < 0.001), and consequently both influence tick burden in a positive proportional direction. Body size was selected as the most appropriate measure of animal's body surface exposed to questing ticks because body mass could be modulated by *ad-libitum* availability of supplementary food. Similar results relating KFI and parasitism in males were obtained for red deer parasitized by *Elaphostrongylus cervi* in south-central Spain (Vicente et al., 2007b), suggesting a close relationship between KFI and macroparasite burden in red deer males in Mediterranean ecosystems. One trait responsible for this male-biased pattern in tick burdens could be related to resource allocation due to mating system, that can be also responsible for the higher relevance of the individual factor explaining tick burden on males than on hinds. This is coherent with results obtained for roe deer, in which sex-biased tick parasitism was only caused by a bias in male and female hunting seasons (Vor et al., 2010; Kiffner et al., 2011), a particular trait that did not account in our study where both sexes were surveyed in every season of the year. In contrast to roe deer males, red deer males defend big harems of several tens of females (Clutton-Brock et al., 1982). Keeping a higher number of hinds away from other males would make red deer males invest more resources in mating than those needed by roe deer males for the same purpose and this may be reflected in the immune system and finally on sex-biased parasitism in red deer males (Vicente et al., 2007b; Corbin et al., 2008). Likely, the apparent absence of any effect of KFI in the model for hinds could be related to the fact that KFI is significantly higher in hinds than in males and it remains constant throughout the year (Santos et al., 2013). Finally, the inverse relationship between KFI and tick burden in combination to the effect of body size, can explain the increasing trend on tick burden with host age.

Sex-related behavioral differences in the use of feeding and water points that could have led to differences in questing tick encounter rates by males and hinds, were discarded on the basis of a study on habitat selection of sympatric wild ungulates in the study estate (Sicilia, 2011). In this study, no sex-biased selection of feeders and water points were observed during summer—when natural food and water are scarce in our territory. It was also observed that both sexes actively selected shrub nearby feeding and water points during daytime and accessed feeders from dusk to dawn, for which no sex-related differences in daily time spent in different habitats were evidenced. If deer spent most of their daily time in feeding and water points this would have been reflected by higher questing tick abundances in these points. However, data from a monthly year-round survey on questing ticks performed in the study hunting estate (F. Ruiz-Fons, unpublished data) showed that higher questing tick abundances are present in the ecotone between shrub and pasture and not in pasture surrounding feeding and water points, being these results coherent with those from habitat use studies (Sicilia, 2011).

Another individual trait that could rely behind male-biased tick parasitism is innate genetic resistance. Fernández-de-Mera et al. (2009a)—in the same study red deer population—found that red deer presenting major histocompatibility complex class II (*MHC-II*) *DRB-2* haplotype 2 displayed significantly higher probability of being lowly parasitized by ticks with respect individuals displaying the other three most abundant *MHC-II-DRB-2* haplotypes in the estate. Data from a second study was re-analyzed for this study and showed that haplotype 2 was more frequent in hinds than in males (Fernández-de-Mera et al., 2009b), which could relate to the male-biased parasitism observed in our study. This hypothesis should be targeted in future experimental and field studies to properly identify its influence on male-biased tick parasitism.

### Host population density driving tick parasitism in red deer

Density of hosts was selected in the independent models for each sex as related to tick burden, probably due to the fact that host densities regulate the percentage of adult ticks in the population that find a host and reproduce, thus contributing to densities of host-seeking ticks (Ruiz-Fons et al., 2012). Host population factor was able to explain a much higher amount of variation in the model for males than in the model for hinds. Likely these findings are again related to behavioral differences between sexes. Hinds live in groups and group size depends in a higher extent on antipredatory behavior—hunting resembles predation in our study population—than of the population density (Jedrzejewski et al., 2006), also in Mediterranean environments (Soriguer et al., 1994). Thus, population density may not be a key factor in determining tick transmission rates in hinds. In contrast, males are more solitary than females (Clutton-Brock et al., 1982), and the contacts in males should be closely dependent of the population density, mainly in the rut season (Carranza et al., 1996).

Wild boar are efficient hosts for *Hyalomma* spp. ticks (Ruiz-Fons et al., 2006), which was evidenced by the positive residual effect of wild boar counts two years prior to survey on tick burdens in both sexes. This residual effect could be perhaps related to the lower abundance of wild boar with respect red deer in the study hunting estate that would make wild boar not to be very relevant in maintaining questing tick abundance.

### Environmental factor driving tick parasitism in red deer

Environmental factor captured most of the variation explained in tick burdens in individual models, especially in hinds (Table 5; Figure 1). Climate modulates both tick activity and survival during their off-host period (Estrada-Peña et al., 2011; Ruiz-Fons et al., 2012) and modulates host-seeking tick abundance. Environmental tick abundance seems to be related to tick burdens in hinds and contribute together with host population and host individual traits to tick burdens in males (Table 5). The effect of climatic variables, with positive influences of average temperature and AET and negative influence of precipitation, may be related to the preponderance of the xerophilic *Hy. lusitanicum* in the study estate which peaks in late spring and early autumn when mean temperatures are high.

### Final statement

The higher weight of host individual and host population factors in the model for males show that intrinsic deer factors are more efficient predictors of tick burden than environmental host-seeking tick abundance, at least when food availability is not a constraint. According to these results, controlling ticks in males such as acaricide spread on males through selective feeders or application of anti-tick vaccines to males only, would hypothetically result in a reduction of tick burdens in hinds since host-seeking tick abundance would be reduced significantly. Whether such an specific tick control measure on males would result in an immediate increase of tick burdens on hinds or in a substantial reduction should be specifically tested in the future.

### Conflict of interest statement

The authors declare that the research was conducted in the absence of any commercial or financial relationships that could be construed as a potential conflict of interest.
